# Therapeutic potential of nasal kisspeptin-54 for reducing α-synuclein accumulation and restoring hippocampal synaptic plasticity in a 6-hydroxydopamine-induced rat model of Parkinson’s disease

**DOI:** 10.55730/1300-0144.6376

**Published:** 2026-06-19

**Authors:** Ayşegül GEMİCİ SİNEN, Emre SAVRAN, Okyanus Bora GÜLEÇ, Osman SİNEN, Narin DERİN

**Affiliations:** 1 Department of Biophysics, Faculty of Medicine, Akdeniz University, Antalya, Turkiye; 2 Faculty of Medicine, Akdeniz University, Antalya, Turkiye; 3 Department of Physiology, Faculty of Medicine, Akdeniz University, Antalya, Turkiye

**Keywords:** Parkinson’s disease, kisspeptin-54, α-synuclein, hippocampus, synaptic plasticity

## Abstract

**Background/aim:**

Parkinson’s disease (PD) is characterized by progressive degeneration of dopaminergic neurons and pathological aggregation of α-synuclein (α-syn), leading to significant motor and cognitive impairments. Kisspeptin-54 (KP-54), a neuropeptide primarily involved in reproductive regulation, has recently been implicated in neurorestorative processes, with emerging evidence suggesting beneficial effects on locomotor activity and cognitive function. However, its therapeutic potential in PD has not yet been fully investigated. This study aimed to evaluate the therapeutic efficacy of chronic nasal KP-54 administration in a 6-hydroxydopamine (6-OHDA) rat model of PD, focusing on motor and cognitive outcomes, hippocampal synaptic plasticity, and α-syn pathology.

**Materials and methods:**

Adult Sprague–Dawley rats underwent unilateral 6-OHDA injections into the medial forebrain bundle to induce dopaminergic degeneration. Beginning 3 weeks after lesion induction, animals received daily nasal KP-54 or vehicle treatment for an additional 3 weeks. Motor performance was assessed using the catalepsy, beam balance, and open field tests, whereas cognitive function was evaluated using the Y-maze and object location test. Baseline assessments were conducted on day 0 prior to lesion formation. Electrophysiological recordings obtained from the hippocampal CA1 region were used to assess synaptic plasticity. Dopaminergic neuron survival and α-syn accumulation in the substantia nigra and hippocampus were quantified by immunofluorescence analysis.

**Results:**

Chronic nasal KP-54 significantly ameliorated motor and cognitive deficits induced by 6-OHDA. KP-54 restored hippocampal synaptic plasticity, enhanced the survival of tyrosine hydroxylase-positive dopaminergic neurons, and reduced α-syn accumulation in both the substantia nigra and hippocampus.

**Conclusion:**

The findings indicate that nasal KP-54 administration exerts neurorestorative effects in a 6-OHDA rat model of PD by preserving dopaminergic neurons, improving synaptic function, and attenuating pathological α-syn aggregation. These results position KP-54 as a promising candidate for disease-modifying therapy in Parkinson’s disease.

## Graphical abstract

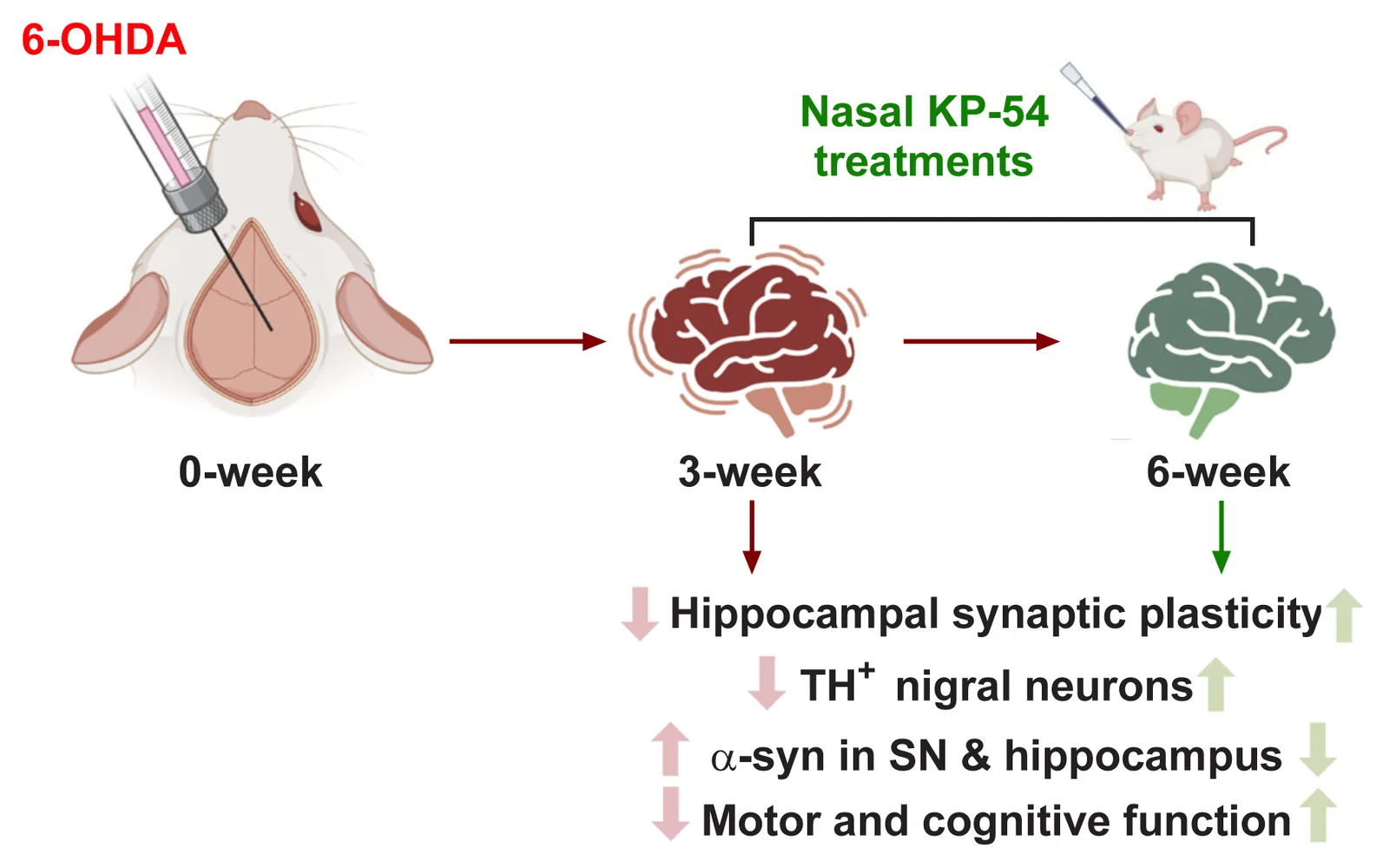

## 1. Introduction

Kisspeptin (KP) is a hypothalamic neuropeptide derived from the *Kiss1* gene, which was initially identified for its metastasis-suppressing properties and originally named metastin. KP exerts multiple physiological functions through binding to the orphan G protein-coupled receptor 54 (GPR54) [ 1,2 ]. Upon binding to GPR54, KP primarily activates the Gq/phospholipase C/inositol 1,4,5-triphosphate signaling cascade, leading to intracellular calcium mobilization. In addition, GPR54 activation has been shown to stimulate protein kinase C, extracellular signal-regulated kinase (ERK), and phosphatidylinositol 3-kinase (PI3K)/Akt signaling pathways, which are involved in neuronal survival, synaptic transmission, and synaptic plasticity [ 3–5]. Recent evidence further suggests that GPR54 activation may increase intracellular cAMP levels, indicating potential crosstalk between Gq-mediated and Gs-mediated signaling mechanisms. Given the critical role of calcium-dependent signaling, ERK phosphorylation, and Akt activation in regulating synaptic plasticity, neuronal survival, and memory formation, these signaling pathways are considered key molecular mediators of neuronal function and plasticity [4,5 ]. Accordingly, downstream molecular events such as intracellular calcium flux, inositol monophosphate formation, and ERK phosphorylation are commonly used as indicators of GPR54 signaling activity [ 4]. The KP preproprotein, composed of 145 amino acids, is enzymatically cleaved into several biologically active fragments (KP-54, KP-14, KP-13, and KP-10), which, despite their varying lengths, share a conserved 10-amino acid C-terminal sequence that confers comparable receptor affinity and biological potency at GPR54 [6]. Subsequent research has revealed that KPs represent a family of peptides with pivotal roles in various physiological processes, most notably in the regulation of metabolism and reproduction [7,8 ]. In addition to its established role in reproduction, KP has been increasingly implicated in a range of physiological processes, including the modulation of locomotor activity [ 9], mood-related behavior [10,11 ], and cognitive function. In particular, KP has been associated with learning and memory, possibly through its actions within key brain regions, including the hippocampus and prefrontal cortex [ 12,13 ].

Parkinson’s disease (PD) is marked by the progressive degeneration of dopaminergic neurons in the substantia nigra pars compacta (SNpc), resulting in striatal dopamine depletion that underlies its characteristic motor symptoms [ 14,15 ]. Although historically viewed as a motor disorder, a wide range of nonmotor symptoms, including those affecting cognition, often emerge early and may even precede motor dysfunction [ 16–18]. Cognitive decline, particularly in executive functions and working memory, can manifest in the early stages of the disease and may advance to dementia as the condition progresses [14,19 ]. The accumulation of intracellular protein aggregates, predominantly composed of α-synuclein (α-syn), represents a key pathological hallmark of PD [ 20,21]. Among the neuropathological changes associated with cognitive decline in PD, early dysfunction of the frontostriatal dopaminergic system and the progressive propagation of α-syn aggregates to limbic and cortical regions are of particular significance [14,22 ]. The hippocampus, a central component of the limbic system, is classically implicated in learning and memory and is also involved in emotion regulation, decision-making, and reward-related processes through its extensive connections with other limbic structures [ 23,24 ]. Numerous functional and structural neuroimaging studies have demonstrated associations of hippocampal hypometabolism and atrophy with memory deficits and progression to dementia in individuals with PD [ 25].

Previous research from our group has shown that KP-54 exerts protective effects in hemiparkinsonian rats, alleviating both motor impairments and nonmotor symptoms, including mood disturbances [9,11 ]. In the present study, we investigated for the first time the therapeutic effects of KP-54 on both motor and nonmotor manifestations of PD, as well as its impact on α-syn accumulation. Additionally, studies by Simon et al. [ 26,27 ] have demonstrated that KP-10 reduces α-syn-induced toxicity and alleviates mitochondrial apoptosis in SH-SY5Y-derived neurons, highlighting its potential neuroprotective effects in models of PD. These findings prompted the hypothesis that KP-54 may have therapeutic potential for ameliorating PD-related cognitive impairments by modulating the underlying electrophysiological mechanisms. The aim of this study was to investigate whether chronic nasal KP-54 administration could ameliorate cognitive impairments in a 6-hydroxydopamine (6-OHDA)-induced rat model of PD.

## 2. Materials and methods

### 2.1. Animals

A total of 24 adult male Sprague–Dawley rats (3 months old) were used in this study. The animals were housed in a temperature-controlled environment (22 ± 1 °C) under a 12-h light/12-h dark cycle, with free access to standard laboratory chow and water. Prior to the experimental procedures, all rats underwent a 1-week handling period to ensure adaptation to the experimental conditions. All animal procedures were reviewed and approved by the Akdeniz University Local Ethics Committee for Animal Experiments (approval no. 30/2025.05.B.001).

### 2.2. Experimental design

**Figure 1:**
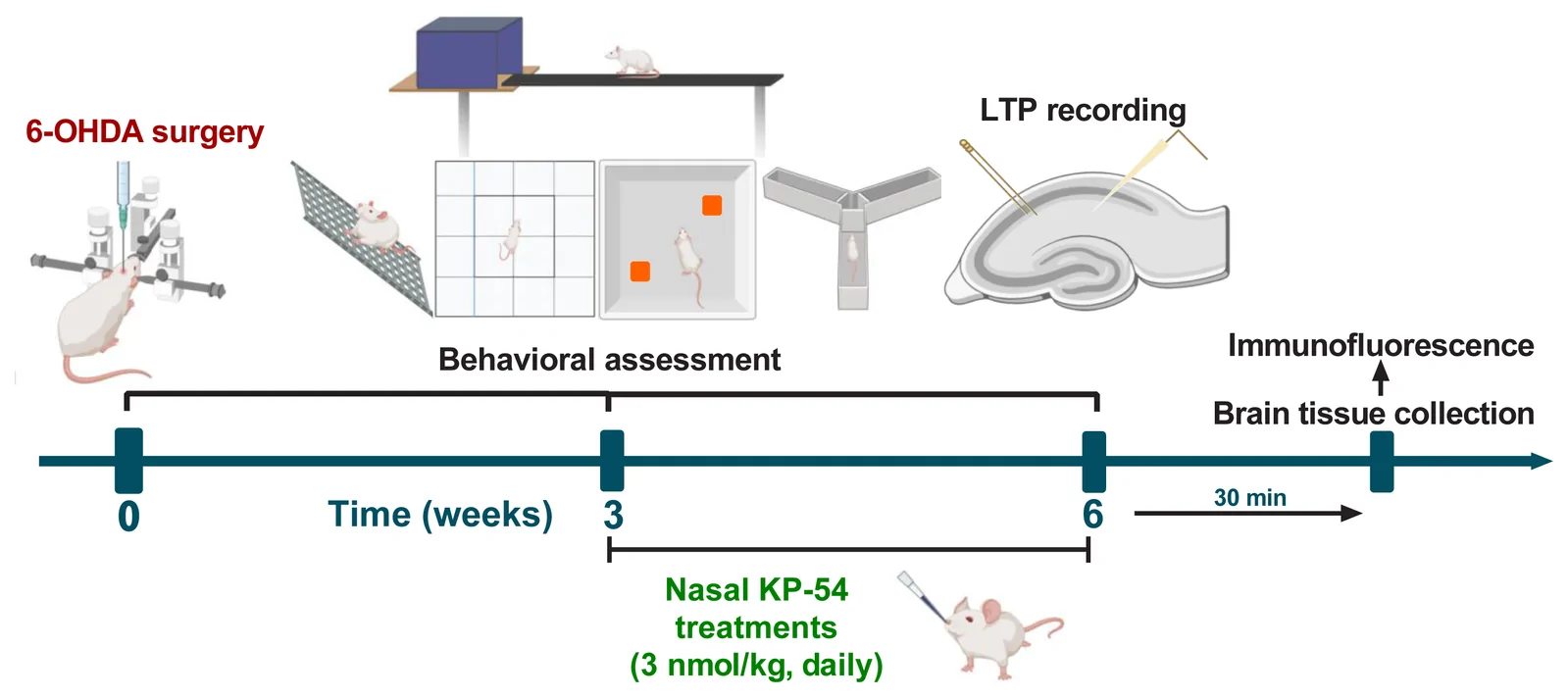
Schematic representation of the experimental timeline created using BioRender (BioRender, Toronto, ON, Canada).

On day 0, all animals were subjected to a battery of behavioral assessments to evaluate baseline motor and cognitive function. Motor performance was assessed using the catalepsy, beam balance, and open field tests, whereas cognitive performance was evaluated using the Y-maze test and the object location test (OLT). Following baseline assessments, animals were randomly assigned to one of three experimental groups: sham (vehicle), 6-OHDA, and 6-OHDA + KP-54. In the 6-OHDA and 6-OHDA + KP-54 groups, the experimental PD rat model was induced by unilateral injection of 6-hydroxydopamine (6-OHDA) into the medial forebrain bundle, whereas the sham group received vehicle solution as a control. After lesion induction, a 3-week period was allowed for stable hemiparkinsonian symptoms to develop. Subsequently, from weeks 3 to 6, animals in the 6-OHDA + KP-54 group received daily nasal administration of KP-54 (3 nmol/kg/day) for 3 weeks to evaluate its therapeutic effects. The selected dose of KP-54 was based on our previous study demonstrating its biological activity in rodent models [ 9]. nasal administration was chosen as a noninvasive route of delivery that facilitates central nervous system exposure while avoiding more invasive procedures. Previous studies have shown that nasally administered KP-54 reaches the brain and produces effects comparable to those observed following central administration [9]. Treatment was initiated 3 weeks after 6-OHDA lesion formation, as previous studies have demonstrated that nigrostriatal degeneration is well established during this period [28]. To assess the progression of motor and cognitive impairments, behavioral tests were repeated on day 21 (week 3) and day 42 (week 6). In week 6, following the final behavioral testing session, hippocampal cornu ammonis 1 (CA1) long-term potentiation (LTP) recordings were performed to evaluate synaptic plasticity. In a separate cohort of animals, immunofluorescence analyses were conducted to assess neurodegeneration and α-syn accumulation. The immunostaining protocol included α-syn in both the substantia nigra (SN) and the hippocampus, whereas tyrosine hydroxylase (TH) immunostaining was performed only in the SN. This design allowed for a comprehensive assessment of KP-54’s effects over time and at the endpoint of the study. The experimental design is shown in Figure 1.

### 2.3. Surgery

Rats were anesthetized with a ketamine/xylazine mixture (50/10 mg/kg, intraperitoneally) and placed in a stereotaxic apparatus equipped with a heating pad to maintain body temperature. After a midline scalp incision, the connective tissue and muscles were retracted to expose the skull, and the bregma was identified. A selective dopaminergic neurotoxin, 6-OHDA (12 μg/μL), was prepared in saline containing 0.1% ascorbic acid. A total volume of 3 μL (3 × 4 μg/μL) was unilaterally injected into the medial forebrain bundle using the following stereotaxic coordinates relative to the bregma: anteroposterior (AP), −2.2 mm; mediolateral (ML), −1.5 mm; dorsoventral (DV), −8.0 mm. The injection was performed with a 30-gauge needle connected to a 10-cm polyethylene tube attached to a Hamilton syringe (Hamilton Company, Reno, NV, USA). Following the injection, the needle was left in place for an additional 3 min to facilitate diffusion of the neurotoxin, and the incision site was closed with silk sutures. Postoperative analgesia was achieved by administering meloxicam intraperitoneally at a dose of 1 mg/kg/day for 2 consecutive days. This procedure is well established for generating an experimental PD model in rats [9,11 ]. Drug treatment was initiated at the beginning of week 3 and continued for 3 weeks. KP-54, dissolved in phosphate-buffered saline, was administered nasally at a dose of 3 nmol/kg/day. For nasal administration, animals were placed in a supine position, and a total volume of 10 μL (5 μL per nostril) of the drug solution was administered using a micropipette.

### 2.4. Behavioral tests

#### 2.4.1. Catalepsy test

Cataleptic behavior was assessed using a vertical wire mesh apparatus (56.5 cm × 23.5 cm; mesh size: 1 cm × 1 cm; wire diameter: 2 mm). Rats were placed on the mesh with all four paws in contact with the surface. The latency to initiate movement, defined as the time elapsed until at least one paw was voluntarily removed from the mesh, was recorded as the catalepsy score. This procedure was conducted in accordance with previously established protocols [ 9,29 ].

#### 2.4.2. Beam balance test

The beam balance test (BBT) is a well-established method for evaluating motor coordination and balance in rodents, particularly rats. The apparatus consisted of a metal beam measuring 60 cm in length and 1.5 cm in width, elevated 90 cm above the ground, with a starting platform at one end and a closed goal box at the other. A padded surface was placed beneath the beam to ensure animal safety and to prevent injury in the event of a fall. Habituation to the apparatus was performed 1 day prior to testing, during which rats were allowed to explore the apparatus for 60 s. During the test session, each rat was placed on the starting platform and allowed to traverse the beam. Performance parameters, including traversal time, the number of paw slips, posture, and stability, were recorded using a digital video tracking system. After a brief rest period (1–3 min), each animal performed three trials. The mean score across the three trials was calculated and used for statistical analysis [30].

#### 2.4.3. Open field test

Locomotor activity was assessed in a square open-field arena (80 cm × 80 cm × 40 cm) constructed of black Plexiglas and divided into 16 equal squares. Each rat was individually placed in the center of the arena, and its movements were recorded for 5 min using a digital video tracking system (Noldus EthoVision XT; Noldus Information Technology, Wageningen, The Netherlands). After each trial, the floor and walls of the arena were thoroughly cleaned with 70% ethanol to eliminate olfactory cues. The total distance traveled and mean velocity were analyzed for each animal [ 9,31 ].

#### 2.4.4. Y-maze test

The Y-maze test was conducted using a three-arm maze, with each arm measuring 50 cm in length, 10 cm in width, and 20 cm in height and positioned at 120° relative to one another. The testing room was enriched with visual cues on the walls to aid spatial orientation. The arms of the maze were designated as the “start,” “other,” and “novel” arms. Initially, each rat was placed at the end of the start arm, and the novel arm was kept closed. Rats were allowed to freely explore the two accessible arms for 10 min. Following this phase, animals were returned to their home cages, and the maze was thoroughly cleaned with 70% ethanol to eliminate olfactory cues. Following a 1-day interval, the novel arm was opened, and each rat was reintroduced to the start arm and allowed to explore all three arms freely for 5 min. During this session, the rats’ behavior was recorded using the Noldus EthoVision XT video tracking system (Noldus Information Technology, Wageningen, The Netherlands). The number of novel arm entries and the time spent in the novel arm were calculated and used as indicators of spatial recognition memory [ 32].

#### 2.4.5. Object location test

The object location test (OLT) was used to evaluate spatial recognition memory in rats. During the training phase, two identical objects were placed in fixed positions within an open-field arena (80 cm × 80 cm × 40 cm), and each rat was allowed to explore them freely for 5 min. The arena was cleaned with 70% ethanol between trials to eliminate olfactory cues. In the retention phase, conducted 1 day later, one of the objects was repositioned to a novel location. Rats were again allowed to explore the arena for 5 min, while their behavior was recorded. Increased exploration time of the relocated object was interpreted as a measure of spatial recognition memory. The discrimination index (DI) was calculated for analysis [33].

### 2.5. Electrophysiological recording

Immediately following the completion of the behavioral and cognitive assessments, animals were anesthetized with urethane (1.25 g/kg, intraperitoneally) and secured in a stereotaxic apparatus (Kopf Instruments, Tujunga, CA, USA) positioned inside a Faraday cage. All procedures were carried out on a heated pad to prevent hypothermia. After the scalp incision, the skull was cleaned using saline-moistened cotton, and the bregma was exposed. Stereotaxic coordinates were determined relative to the bregma. Small burr holes (approximately 0.5 mm in diameter) were drilled using a precision drill (RWD Life Science, San Diego, CA, USA) to allow electrode placement into the medial Schaffer collaterals (AP: −3.1 mm, ML: 3.1 mm) and the CA1 region (AP: −2.8 mm, ML: 1.8 mm). A reference screw electrode (#0–80, 1/8-inch stainless steel) was implanted approximately 3 mm posterior to lambda, with gentle contact with the cortical surface. A silver chloride disc electrode was attached to the tail and used as the ground electrode. A bipolar stimulating electrode (platinum–iridium, stainless steel, 0.010-inch diameter; Plastic One, Roanoke, VA, USA) was inserted 2.7 mm below the cortical surface into the Schaffer collaterals via the designated burr hole and adjusted using a micromanipulator. The electrode was connected via low-impedance wires to a stimulus isolator (MASTER-9 with ISO-Flex; A.M.P.I., Jerusalem, Israel). A monopolar stainless steel recording electrode (0.005 inch; Plastic One, Roanoke, VA, USA) was implanted in the CA1 region and connected to a differential electrophysiological amplifier (Bio Amp; ADInstruments, Sydney, Australia; band-pass filter: 1 Hz–3 kHz). The reference and ground electrodes were also connected to the amplifier. Data acquisition and visualization were conducted using Scope software (ADInstruments, Sydney, Australia). Both the stimulating and recording electrodes were gradually lowered until a characteristic electrophysiological response was recorded from the hippocampal CA1 region. This response consisted of a positive deflection corresponding to the maximal field excitatory postsynaptic potential (fEPSP), followed by a negative population spike (PS). Electrodes were advanced in 0.1 mm increments until the maximal response was obtained. The final depth of the recording electrode typically ranged between 2.7 and 3.2 mm. Optimal ventral positioning was confirmed by observing evoked fEPSPs in the CA1 region following Schaffer collateral stimulation, as previously described [34,35 ].

Baseline recordings were obtained at a frequency of 0.1 Hz using biphasic square-wave pulses of 0.1 ms duration. To generate the input–output (I/O) curve, stimulation commenced 15 min after electrode placement, using 175-ms trains of monophasic pulses delivered every 20 s. Stimulus intensities ranged from 0.1 to 1.5 mA. For each intensity, the mean response of three consecutive evoked potentials was calculated. The relationship between stimulus intensity and the fEPSP slope or amplitude was fitted to a sigmoidal curve, from which the stimulus intensity eliciting 50% of the maximal response was determined and used as the test stimulus intensity. For experimental animals, this test intensity ranged between 0.15 and 0.6 mA, and it was maintained throughout the electrophysiological recordings [ 31,34 ].

### 2.6. Immunofluorescence analysis

In each experimental group, eight animals were used for behavioral assessments; thereafter, five animals per group were allocated to hippocampal LTP recordings, whereas the remaining three animals per group were processed for immunofluorescence analyses. Following transcardiac perfusion with 50 mL of saline, brains were fixed in 4% paraformaldehyde and cryoprotected in 20% sucrose for 24 h. Coronal sections (40 μm) were obtained using a cryostat (CM1850; Leica Microsystems, Nussloch, Germany) and stored at −20 °C in 0.1 M phosphate buffer containing 30% sucrose and 25% ethylene glycol. On the day of analysis, sections were incubated either with a sheep anti-TH primary antibody (1:1000, Abcam, Cambridge, United Kingdom), followed by a donkey antisheep Alexa Fluor 547 secondary antibody (1:1000; Thermo Fisher Scientific, Waltham, MA, USA), or with rabbit anti-α-syn primary antibody (1:500; Thermo Fisher Scientific, Waltham, MA, USA), followed by a donkey antirabbit Alexa Fluor 488 secondary antibody (1:1000; Thermo Fisher Scientific, Waltham, MA, USA). Fluorescent images were acquired using an Olympus BX43 fluorescence microscope (Olympus Corporation, Tokyo, Japan) equipped with appropriate filter sets. All analyses were performed in a blinded manner. TH-positive and α-syn-positive neurons in the SN were quantified bilaterally across the rostrocaudal axis using three alternating sections per hemisphere, and the mean cell counts were used as representative values for each animal. In hippocampal tissue, fluorescence intensity was quantified using ImageJ software (National Institutes of Health, Bethesda, MD, USA), and relative changes were expressed as a percentage of the control group.

### 2.7. Statistical analysis

The normality of the data distribution was assessed using the Shapiro–Wilk test. For data that followed a normal distribution, a two-way repeated-measures analysis of variance (ANOVA) followed by Tukey’s post hoc test was performed to determine statistical differences among groups. In cases where the data did not meet the assumptions of normality, the Kruskal–Wallis test was applied, followed by Dunn’s post hoc test. A p < 0.05 was considered statistically significant. All statistical analyses were conducted using GraphPad Prism (GraphPad Software, San Diego, CA, USA).

## 3. Results

### 3.1. Chronic nasal KP-54 administration improves 6-OHDA-induced motor deficits

Motor performance assessments demonstrated that, relative to baseline (catalepsy latency: 0.92 ± 0.06 s; BBT score: 5.75 ± 0.16; total distance traveled: 1756.43 ± 99.83 cm; velocity: 6.24 ± 0.46 cm/s), the 6-OHDA group exhibited significant impairments by week 3, including increased catalepsy latency (3.70 ± 0.21 s, p < 0.0001, n = 8), reduced BBT score (2.62 ± 0.53, p < 0.05, n = 8), decreased total distance traveled (630.63 ± 92.21 cm, p < 0.01, n = 8), and reduced velocity (1.96 ± 0.17 cm/s, p < 0.01, n = 8).

While no significant changes were observed in the 6-OHDA group, daily KP-54 treatment from weeks 3 to 6 significantly improved the motor deficits observed at week 3, as evidenced by reduced catalepsy latency (1.69 ± 0.31 s, p < 0.01, n = 8) and increased velocity (3.26 ± 0.41 cm/s, p < 0.05, n = 8). However, the increases in total distance traveled (952.98 ± 110.92 cm, p = 0.12, n = 8) and BBT score (4.75 ± 0.64, p = 0.24, n = 8) did not reach statistical significance.

**Figure 2:**
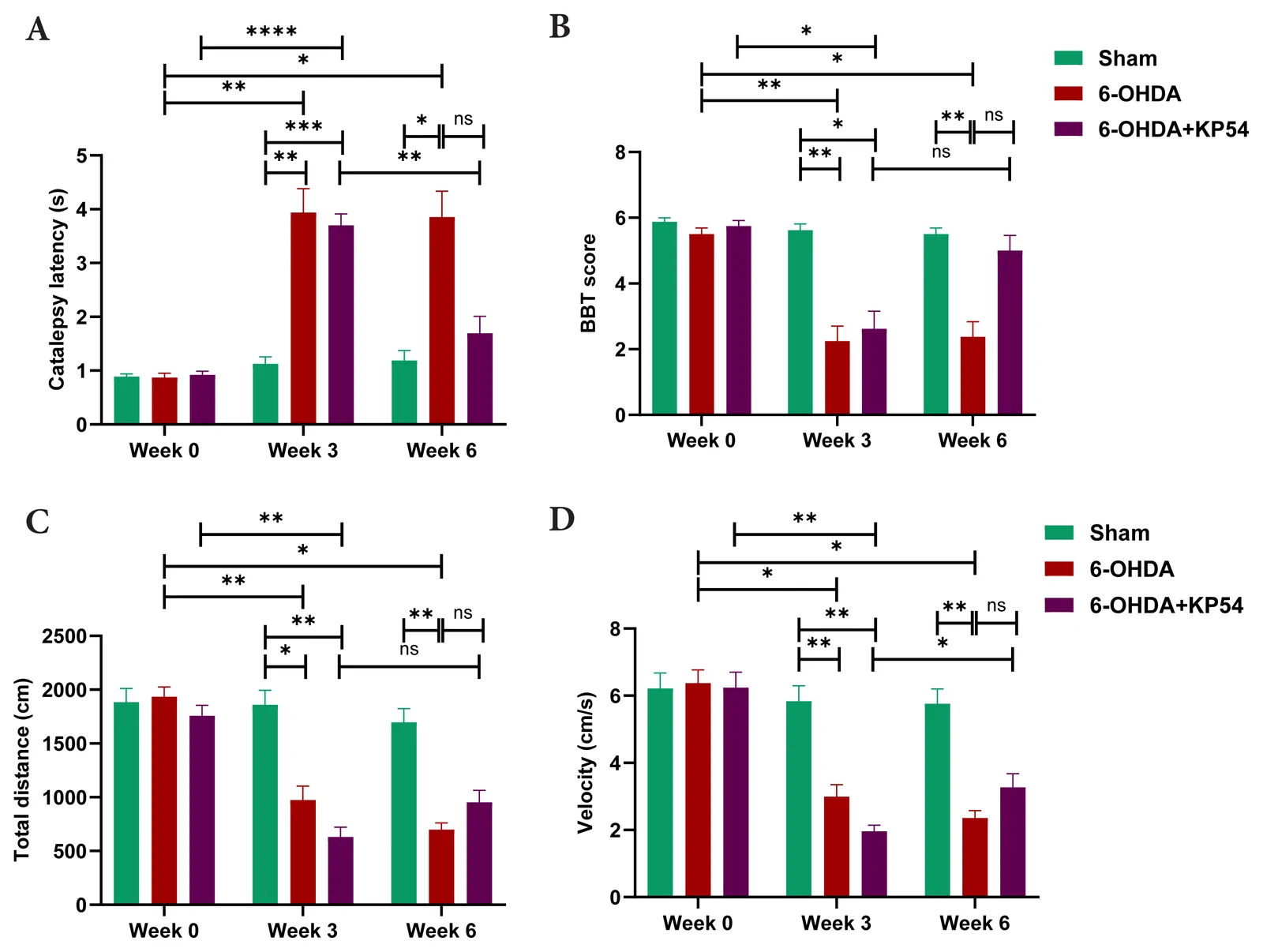
Effects of KP-54 treatment on motor performance in 6-OHDA-lesioned rats. (A) Catalepsy latency, (B) BBT score, (C) total distance traveled, and (D) mean velocity in the open field test. Data are presented as mean ± SEM. Statistical analyses were performed using a two-way repeated-measures ANOVA followed by Tukey’s post hoc test. ns: not significant; n = 8 per group.

At week 6, all motor performance parameters remained significantly impaired in the 6-OHDA group compared with the sham group. When the 6-OHDA and 6-OHDA + KP-54 groups were compared at week 6, KP-54 treatment was associated with consistent reductions in behavioral deficits; however, these improvements did not reach statistical significance (Figure 2).

### 3.2. Chronic nasal KP-54 administration ameliorates 6-OHDA-induced cognitive deficits

**Figure 3:**
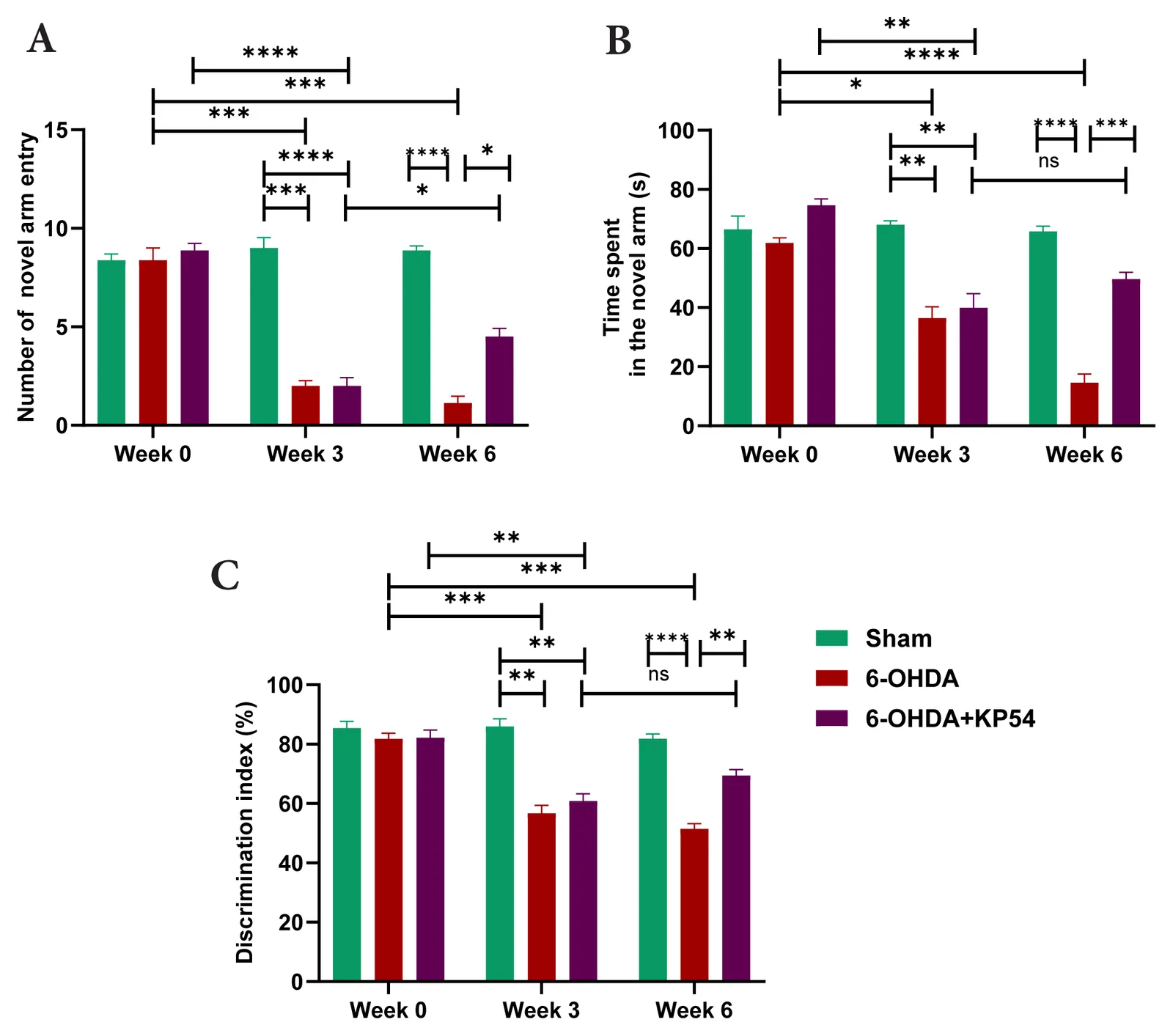
Effects of KP-54 treatment on cognitive performance in 6-OHDA-lesioned rats. (A) Number of novel arm entries, (B) time spent in the novel arm in the Y-maze test, and (C) DI in the OLT. Data are presented as mean ± SEM. Statistical analyses were performed using a two-way repeated-measures ANOVA followed by Tukey’s post hoc test. ns: not significant; n = 8 per group.

Cognitive assessments indicated that, compared with baseline (number of novel arm entries: 8.87 ± 0.35; time spent in the novel arm: 74.63 ± 2.17 s; DI: 82.17% ± 2.65%), the 6-OHDA group exhibited significant impairments by week 3, as evidenced by fewer novel arm entries (2.00 ± 0.42), reduced time spent in the novel arm (39.97 ± 4.78 s), and a lower DI (60.86% ± 2.45 %). Following KP-54 administration (weeks 3–6), animals exhibited a significant increase in novel arm entries (4.5 ± 0.42, p < 0.05, n = 8), whereas the increases in time spent in the novel arm (49.63 ± 2.33 s, p = 0.45, n = 8) and DI (69.49% ± 1.95%, p = 0.23, n = 8) did not reach statistical significance. Furthermore, analysis at week 6 demonstrated significant deficits in the 6-OHDA group compared with the sham group. Although these deficits were attenuated in the 6-OHDA + KP-54 group, the improvement was most evident for novel arm entries (Figure 3).

### 3.3. Chronic nasal KP-54 administration attenuates 6-OHDA-induced impairments in hippocampal synaptic plasticity

**Figure 4:**
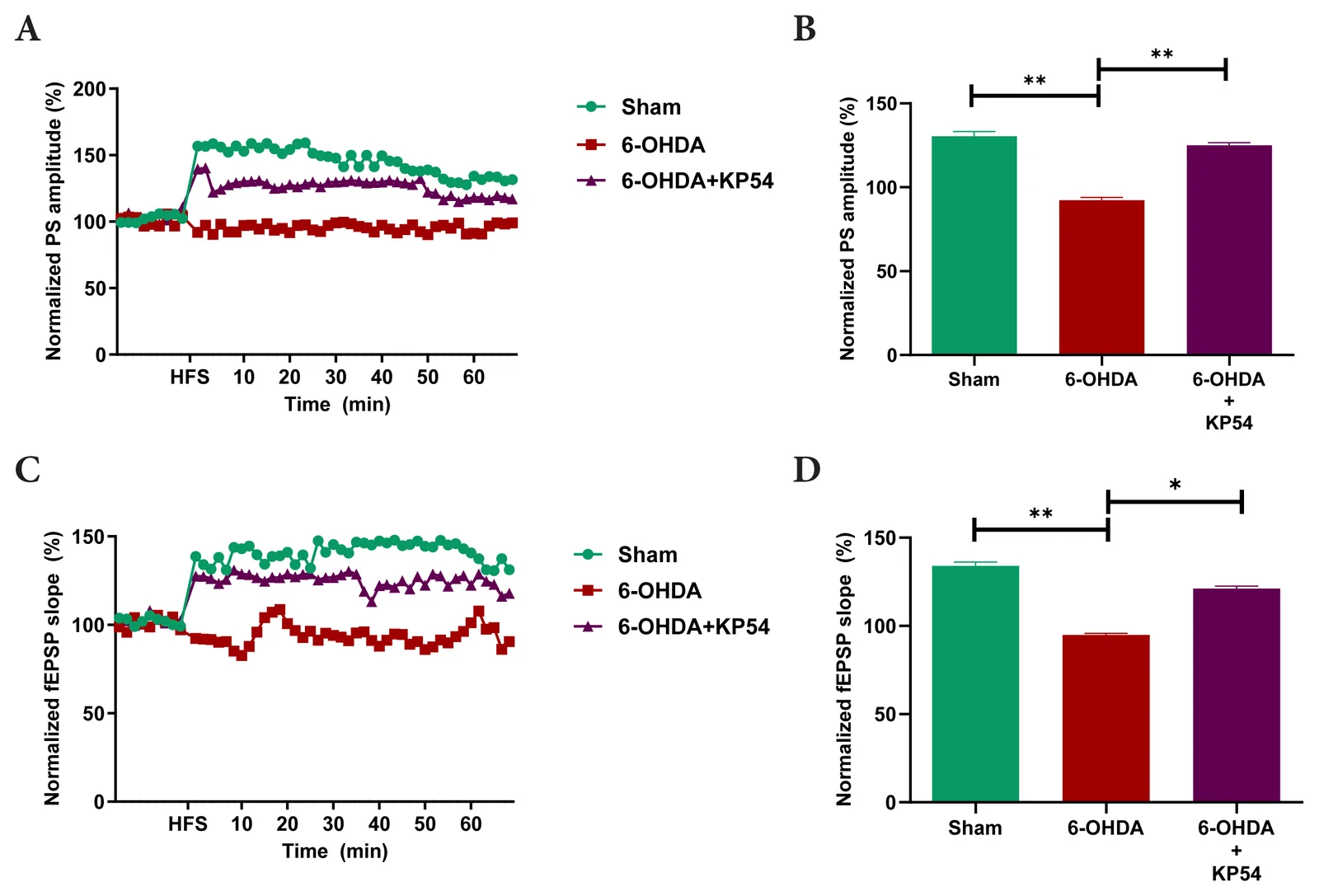
Effects of KP-54 treatment on hippocampal LTP impairments induced by 6-OHDA lesions. Time course of normalized PS amplitude (A) and normalized fEPSP slope (C) following HFS in the sham, 6-OHDA, and 6-OHDA + KP-54 groups. Quantification of mean PS amplitude (B) and mean fEPSP slope (D) during the last 10 min of recordings. Data are presented as mean ± SEM. Statistical analysis was performed using the Kruskal–Wallis test followed by Dunn’s post hoc test. n = 5 per group.

To evaluate the effects of KP-54 on synaptic plasticity, high-frequency stimulation (HFS)-induced LTP was recorded from the hippocampal CA1 region under urethane anesthesia. In the 6-OHDA group, both the PS amplitude (89% ± 1.7%, p < 0.01, n = 5) and the field excitatory postsynaptic potential (fEPSP) slope (91% ± 1.2%, p < 0.01, n = 5) were significantly reduced compared with the sham group (PS: 131% ± 2.7%; fEPSP: 134% ± 2.2%), indicating impaired LTP induction. Nasal administration of KP-54 significantly attenuated these deficits, as evidenced by higher PS amplitudes (124% ± 2%, p < 0.01, n = 5) and fEPSP slopes (121% ± 1.3%, p < 0.05, n = 5) compared with the 6-OHDA group. These findings indicate that KP-54 treatment partially restored hippocampal synaptic plasticity following 6-OHDA lesions (Figure 4).

### 3.4. Chronic nasal KP-54 administration attenuates the loss of TH-positive neurons and reduces α-syn accumulation

**Figure 5:**
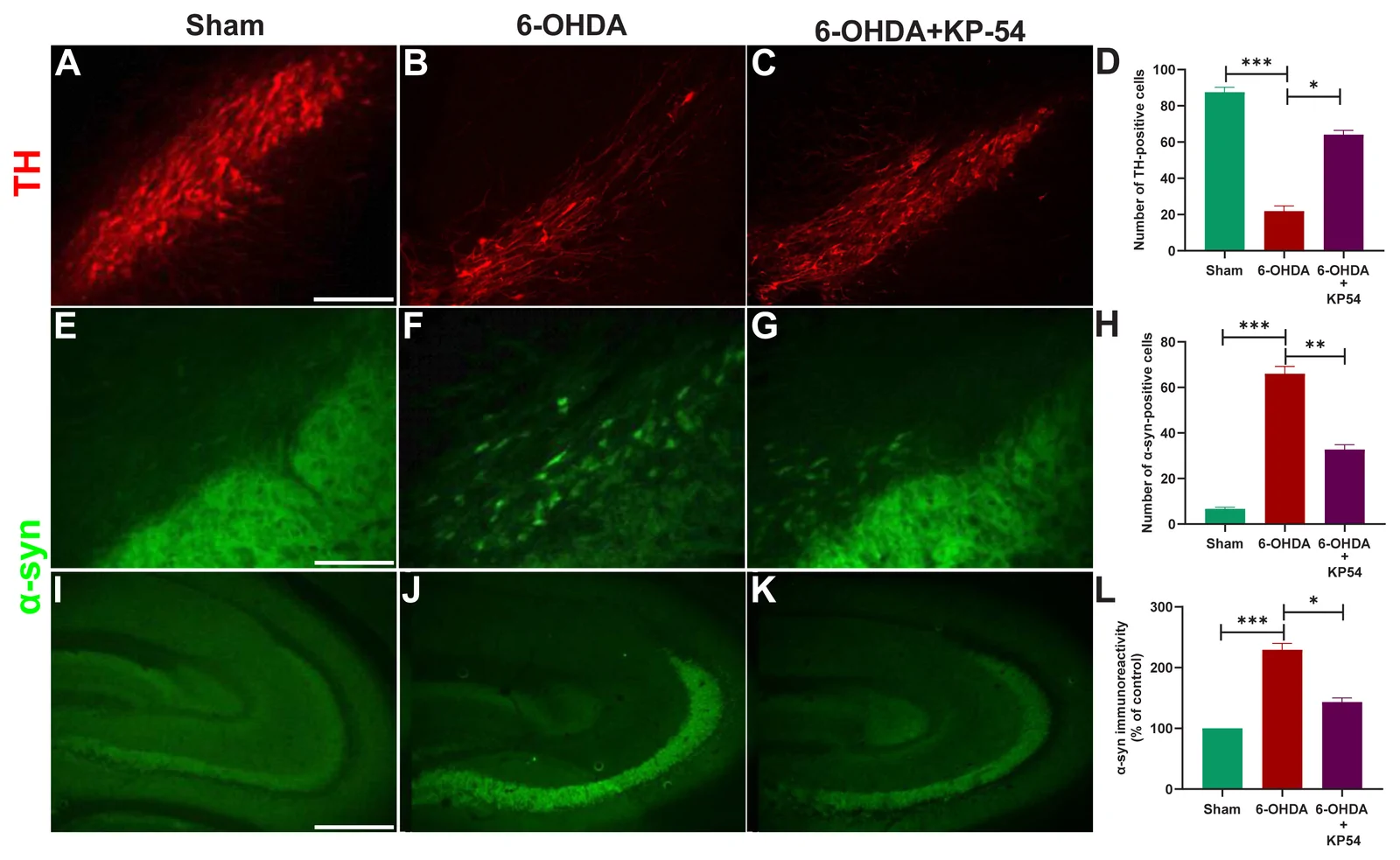
Effects of KP-54 treatment on TH and α-syn immunofluorescence in the 6-OHDA-induced rat model of PD. Representative immunofluorescence images and quantitative analyses of TH-positive neurons in the SN (A–D) and α-syn immunoreactivity in the SN (E–H) and hippocampus (I–L). Data are presented as mean ± SEM. Statistical analysis was performed using the Kruskal–Wallis test followed by Dunn’s post hoc test. n = 3 per group. Scale bars = 100 μm.

Immunofluorescence analysis revealed a marked loss of TH-positive neurons in the SN following 6-OHDA lesioning (21.89 ± 2.78 cells, p < 0.001, n = 3) compared with the sham group (87.56 ± 2.66 cells). Nasal KP-54 administration significantly increased the number of TH-positive neurons (64.11 ± 2.34 cells, p < 0.05, n = 3) compared with the 6-OHDA group. Conversely, α-syn immunostaining demonstrated a marked increase in α-syn-positive neurons in the 6-OHDA group (SN: 66 ± 3.3 cells, p < 0.001; hippocampus: 229% ± 10%, p < 0.001, n = 3), which was significantly reduced following KP-54 treatment in both the SN (33 ± 2.1 cells, p < 0.01, n = 3) and the hippocampus (143% ± 6.7%, p < 0.05, n = 3) (Figure 5). These findings indicate that KP-54 treatment was associated with increased TH-positive neuron counts and reduced α-syn accumulation in the 6-OHDA-induced PD model (Figure 5).

## 4. Discussion

In this study, we demonstrated that chronic nasal KP-54 administration ameliorated motor and cognitive deficits in the 6-OHDA-induced rat model of PD. KP-54 partially restored hippocampal synaptic function, as evidenced by increased fEPSP slopes and PS amplitudes following 6-OHDA lesioning. Immunofluorescence analysis further demonstrated reduced α-syn accumulation in both the SN and the hippocampus. Together, these findings suggest that KP-54 may exert dopaminergic neuroprotective effects while reducing pathological α-syn accumulation, highlighting its potential as a therapeutic candidate for PD.

The present findings extend our previous work, suggesting that chronic nasal KP-54 administration may influence not only motor function and nigrostriatal integrity but also cognitive performance and hippocampal synaptic function in the 6-OHDA model of Parkinson’s disease [ 9,11 ]. Improvements in synaptic plasticity and reduced pathological α-syn accumulation in both the SN and hippocampus indicate that KP-54 may act on a broader range of neural processes than previously recognized. Although preliminary, the combined electrophysiological, α-syn, and TH immunofluoresence findings provide initial mechanistic insight into how KP-54 may modulate dopaminergic degeneration, synaptic dysfunction, and α-syn pathology. Further studies with larger cohorts and complementary approaches are needed to confirm these effects and assess their translational relevance. Overall, these findings provide a more comprehensive understanding of the potential neuroprotective and neurorestorative effects of KP-54, supporting further investigation of this peptide as a potential therapeutic candidate for Parkinson’s disease.

Parkinson’s disease is the second most prevalent neurodegenerative disorder after Alzheimer’s disease and is characterized by the progressive loss of dopaminergic neurons in the SNpc and subsequent striatal dopamine depletion, leading to motor symptoms such as tremor, bradykinesia, rigidity, and postural instability [ 19,36 ]. Consistent with these pathological features, chronic nasal KP-54 administration improved motor performance in the 6-OHDA-induced rat model by reducing catalepsy latency, improving balance, and partially restoring locomotor activity. In addition, previous studies from our group demonstrated preservation of TH-positive nigral neurons and striatal dopamine levels following KP-54 treatment [ 9,37 ]. PD is increasingly recognized as a multisystem disorder with nonmotor symptoms, including sensory, autonomic, sleep, neuropsychiatric, and cognitive deficits, often preceding motor onset [ 38,39 ]. Accumulation of α-syn is thought to contribute to both motor and cognitive deficits through dysfunction of the nigrostriatal and limbic–cortical circuits [ 19,40 ]. In line with these observations, KP-54 partially alleviated spatial recognition memory deficits in the OLT and Y-maze test, consistent with a potential role in modulating hippocampal synaptic plasticity and memory through glutamatergic and dopaminergic signaling pathways [ 41,42 ].

KPs, through GPR54 activation, have been shown to exert neuroprotective effects beyond their established role in reproduction by reducing oxidative stress, apoptosis, and functional deficits in experimental models of neurological injury [ 42]. KP-54, the most stable endogenous isoform, has been reported to exert neuroprotective effects in experimental models of cerebral ischemia, Alzheimer’s disease, and subarachnoid hemorrhage, supporting its potential to modulate neuronal survival and cognitive function [42–44]. The widespread expression of KP and GPR54 in the hippocampus and amygdala further supports their modulatory roles in learning and memory [45], suggesting that KP-54 may contribute to the attenuation of memory deficits in experimental models of PD.

Taken together, our findings suggest that KP-54 may mitigate both motor and cognitive dysfunctions in experimental models of Parkinson’s disease, warranting further investigation of its underlying mechanisms and therapeutic potential. In PD, dopaminergic dysfunction affects both motor and cognitive domains and is associated with alterations in hippocampal synaptic plasticity [14,46 ]. Synaptic dysfunction driven by presynaptic α-syn accumulation is an early hallmark of PD, disrupting synaptic proteins, dopamine release, vesicle recycling, and LTP in the striatum, hippocampus, and prefrontal cortex [ 46–48]. In neurotoxic and transgenic models, CA1 LTP is impaired owing to disrupted dopaminergic signaling and altered NMDA receptor subunit composition (NR2A/NR2B). These alterations can be partially restored by L-DOPA through D1/D5 receptor activation [49,50 ]. Similarly, in MPTP models, NMDA receptor-mediated hippocampal fEPSP slopes are reduced by approximately 23%, accompanied by impaired LTP and prolonged LTD [ 51]. These data highlight the dependence of hippocampal plasticity on dopamine and the functional deficits arising from its disruption. Consistent with these observations, KP-54 partially ameliorated hippocampal LTP deficits in the present study. These findings are in agreement with previous evidence showing that KP-13 attenuates amyloid-β–induced hippocampal dysfunction and enhances spatial memory consolidation [52,53 ], supporting a potential role for KP peptides in the regulation of synaptic plasticity across neurodegenerative conditions.

KP-54 also preserved TH-positive neurons in the SN, consistent with improved dopaminergic integrity. As expected, 6-OHDA induced a marked loss of TH-positive neurons [ 54,55 ], whereas KP-54 significantly attenuated this reduction [ 9,42 ]. In parallel, KP-54 reduced α-syn accumulation in both the SN and the hippocampus, two regions critically involved in PD-related motor and cognitive dysfunction [ 19]. This reduction may contribute to the attenuation of neuropathological processes associated with synaptic dysfunction and neuronal loss [47,48 ]. These findings support the continued investigation of KP-54 as a promising therapeutic candidate for PD, with potential neuroprotective and antiproteinopathic effects.

A limitation of this study is that synaptic plasticity was assessed only functionally, without structural or molecular analyses. In addition, the inability to evaluate gene expression due to budgetary constraints constitutes another limitation of the present study. Assessment of gene expression profiles is planned future studies. Future studies should investigate synaptic architecture (e.g., dendritic spine density and synaptic protein markers) together with the expression of LTP-related receptors, particularly AMPA and NMDA receptor subunits, to further elucidate the mechanisms by which KP-54 modulates hippocampal synaptic plasticity. It remains to be determined whether the observed effects of KP-54 involve D1/D5 receptor signaling, NMDA receptor-dependent mechanisms, or downstream neurotrophic signaling pathways. Further studies should also investigate dose-response relationships, long-term safety, and the potential effects of KP-54 on α-syn clearance mechanisms, including autophagy, lysosomal degradation, and mitochondrial function.

## 5. Conclusion

Chronic nasal administration of KP-54 produced multifaceted neuroprotective effects in a 6-OHDA-induced rat model of PD. KP-54 treatment improved lesion-induced motor deficits, as reflected by enhanced performance in behavioral assessments. It also improved spatial recognition memory, as assessed by the Y-maze test and the OLT. It partially restored hippocampal synaptic function, as evidenced by increased fEPSP slopes and PS amplitudes, increased TH-positive neuron counts in the SN, and reduced pathological α-syn accumulation in both the SN and the hippocampus. Together, these findings suggest that KP-54 may exert complementary neuroprotective and antiproteinopathic effects, thereby supporting dopaminergic neuronal integrity and reducing pathological α-syn accumulation, which may contribute to improvements in both motor and cognitive function in this preclinical model of PD.

## Data Availability

The data supporting the findings of this study are available from the corresponding author upon reasonable request.
